# Supplementary material for: Generation of isogenic models of Angelman syndrome and Prader-Willi syndrome in CRISPR/Cas9-engineered human embryonic stem cells
Source: PLoS One. 2024 Nov 1;19(11):e0311565. doi: 10.1371/journal.pone.0311565 (PMC11530062; doi:10.1371/journal.pone.0311565)
Supplement: S1 Fig — Expression of UBE3A is set to a relative value of 1 for the wild type parental H9 line. As later determined through confirmatory testing, H9Δmat15q_1 is designated as Tfx1_Clone13, H9Δmat15q_2 is designated as Tfx4_Clone22, H9Δpat15q is designated as Tfx4_Clone2. (PDF) [file pone.0311565.s001.pdf]

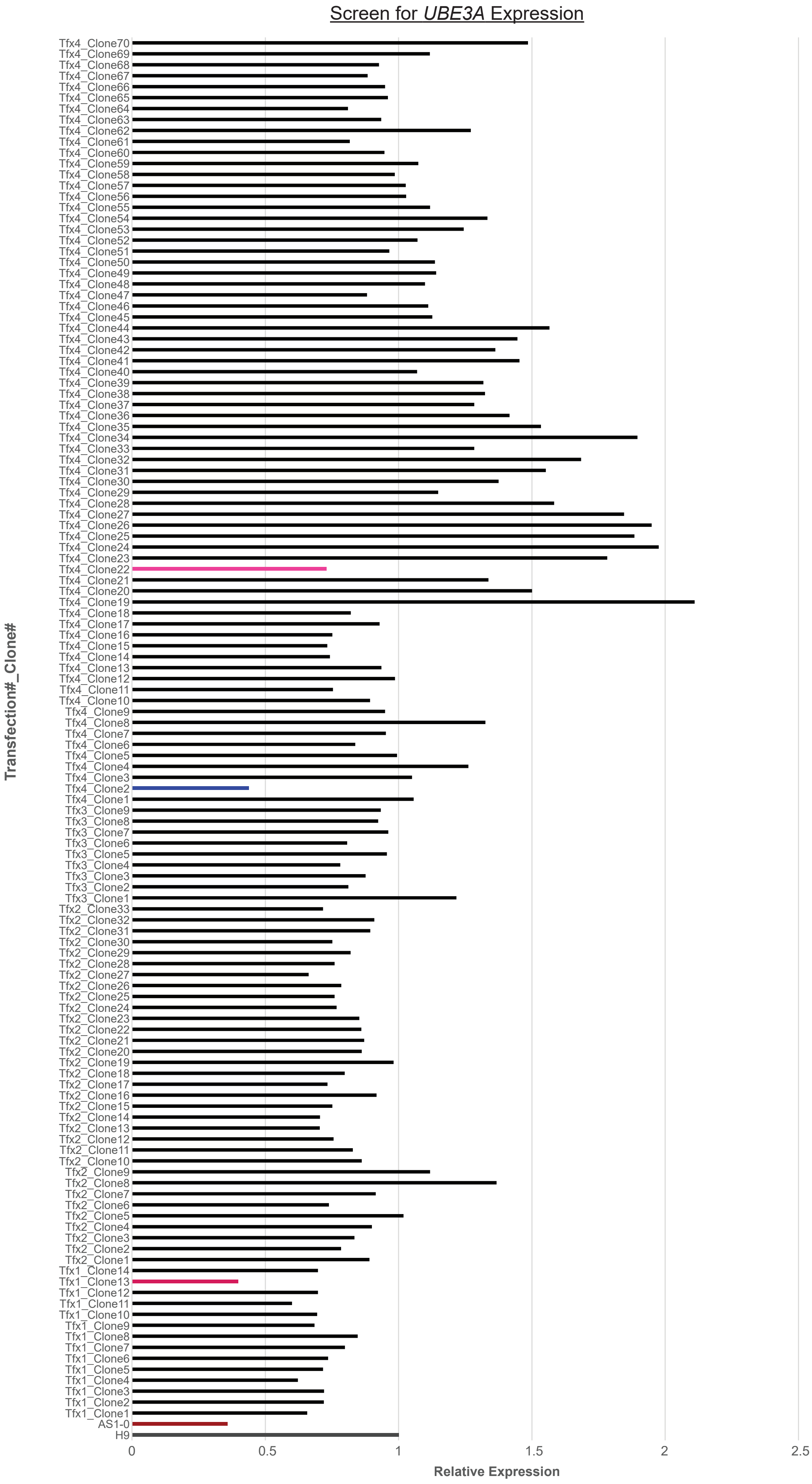

Supplemental Figure 1. Compilation of screening data from *UBE3A* expression from all clones from all transfections. Relative expression of *UBE3A* is set to a relative value of 1 for the wild type parental H9 line. H9Δmat15q_1 is designated as Tfx1_Clone13, H9Δmat15q_2 is designated as Tfx4_Clone22, H9Δpat15q is designated as Tfx4_Clone2.
