# Supplementary material for: Generation of isogenic models of Angelman syndrome and Prader-Willi syndrome in CRISPR/Cas9-engineered human embryonic stem cells
Source: PLoS One. 2024 Nov 1;19(11):e0311565. doi: 10.1371/journal.pone.0311565 (PMC11530062; doi:10.1371/journal.pone.0311565)
Supplement: S2 Fig — Plasmid map of PX459 containing guide RNA targeting GOLGA8 repeat sequences. Primers for individual amplicons are indicated on the map. The plasmid was used as a positive control for PCR reactions 1 through 3. An additional amplicon for the TBX5 promoter was used as a positive control for genomic DNA for each PCR reaction (number 4). For each of the lines, including the parental H9 line, the TBX5 amplicon amplified. The PX459 amplicons were detected from purified plasmid DNA but failed to amplify from any genomic DNA sample from edited clones. (PDF) [file pone.0311565.s002.pdf]

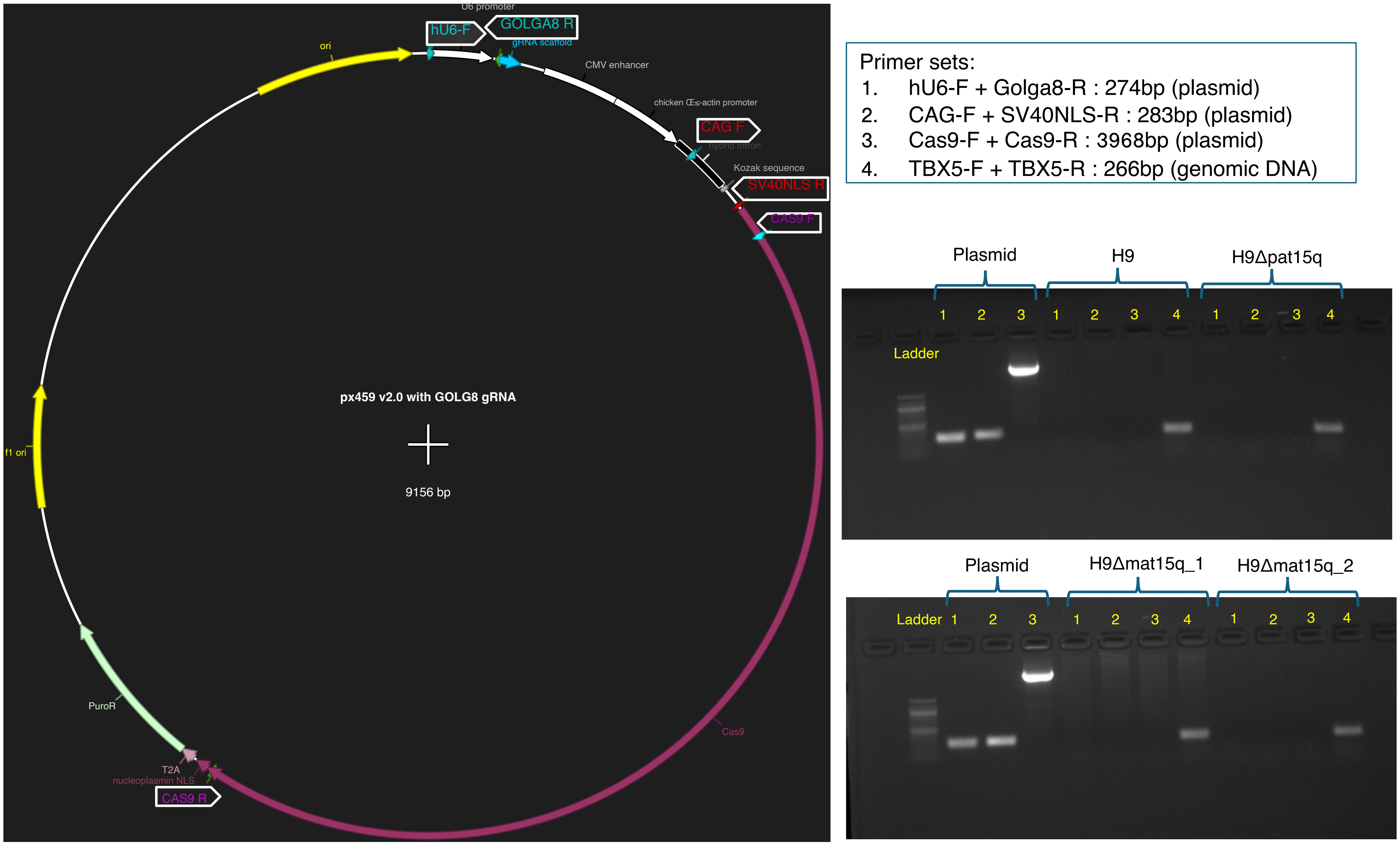

Supplemental Figure 2. Screen for integration of PX459 GOLGA8 guide RNA plasmid. Plasmid map of PX459 containing guide RNA targeting GOLGA8 repeat sequences. Primers for individual amplicons are indicated on the map. The plasmid was used as a positive control for PCR reactions 1 through 3. An additional amplicon for the TBX5 promoter was used as a positive control for genomic DNA for each PCR reaction (number 4). For each of the lines, including the parental H9 line, the TBX5 amplicon amplified. The PX459 amplicons were detected from purified plasmid DNA but failed to amplify from any genomic DNA sample from edited clones.
