# Supplementary material for: Generation of isogenic models of Angelman syndrome and Prader-Willi syndrome in CRISPR/Cas9-engineered human embryonic stem cells
Source: PLoS One. 2024 Nov 1;19(11):e0311565. doi: 10.1371/journal.pone.0311565 (PMC11530062; doi:10.1371/journal.pone.0311565)
Supplement: S5 Fig — A) Immunocytochemistry for Oct4 with nuclear marker DAPI. Images taken at 20X and 63X. Scale bars equal 50 μm and 25 μm, respectively. B) qPCR analysis of GABRG3 in hESCs (n = 3 biological replicates). RNA expression is presented relative to the parental H9 line. Error bars represent relative min/max calculated with error propagation. Statistical analysis was performed using a one-way ANOVA followed by Dunnett’s test. Significance is reported as the results of Dunnett’s test. * = p < 0.05. (PDF) [file pone.0311565.s005.pdf]

A) Immunocytochemistry in H9Δmat15q_2 ESCs

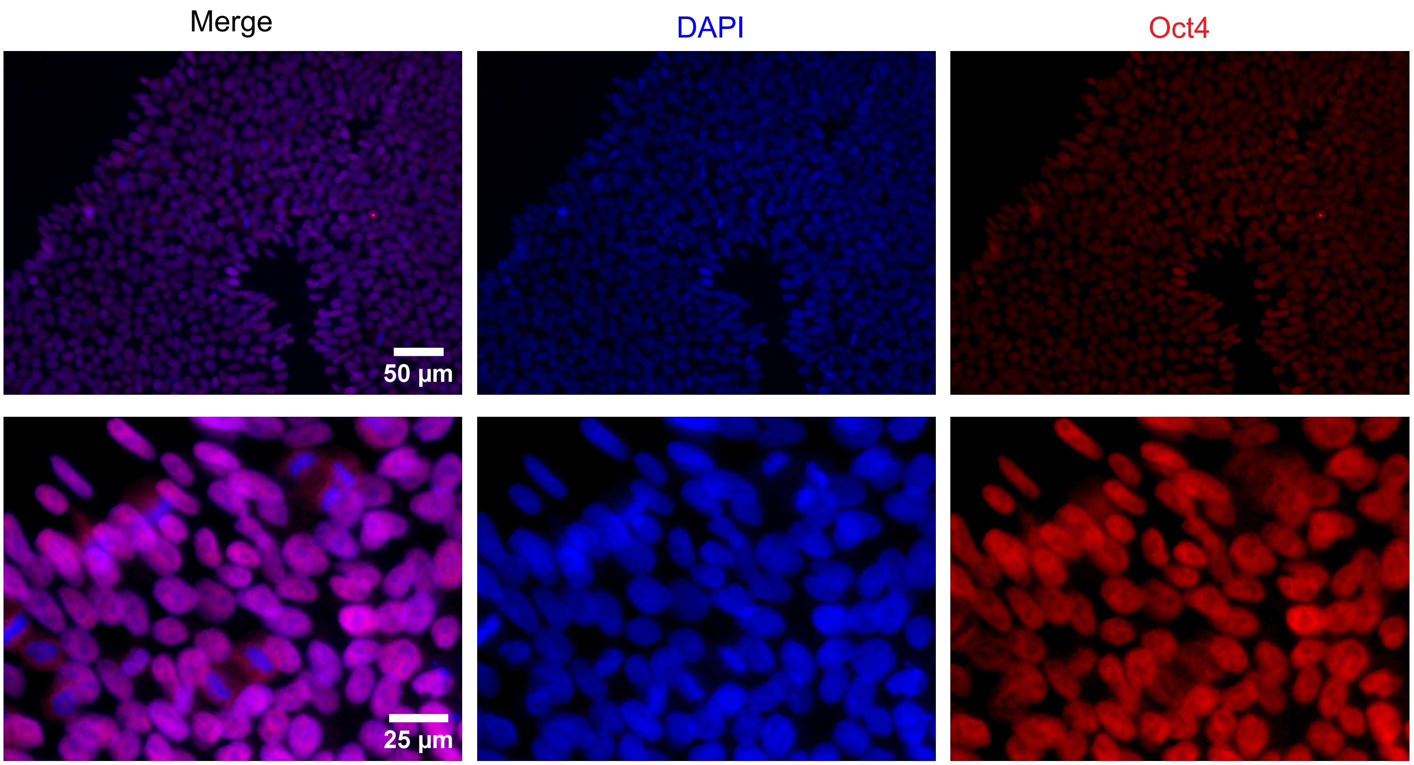

B) *GABRG3* Expression in hESCs

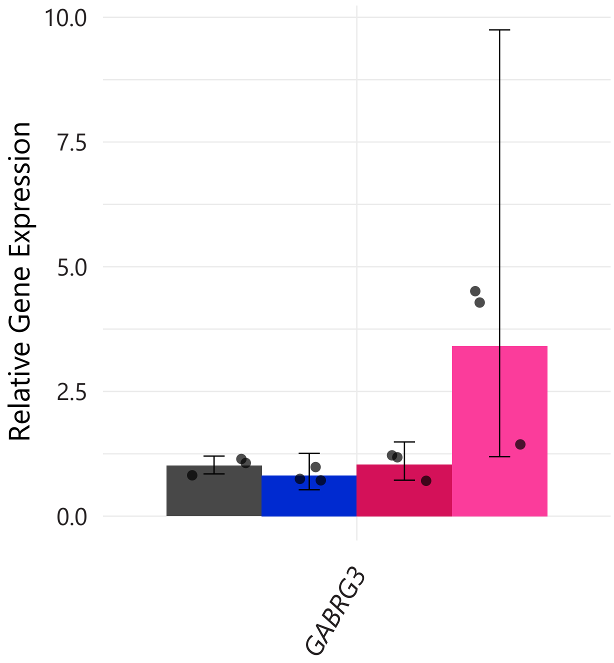

Supplemental Figure 5. Further characterization of hESCs. A) Immunocytochemistry for Oct4 with nuclear marker DAPI. Images taken at 20X and 63X. Scale bars equal 50 µm and 25 µm, respectively. B) qPCR analysis of GABRG3 in hESCs (n = 3 biological replicates). RNA expression is presented relative to the parental H9 line. Error bars represent relative min/max calculated with error propagation. Statistical analysis was performed using a one-way ANOVA followed by Dunnett's test. Significance is reported as the results of Dunnett's test. * = $p < 0.05$.
