# Supplementary material for: Generation of isogenic models of Angelman syndrome and Prader-Willi syndrome in CRISPR/Cas9-engineered human embryonic stem cells
Source: PLoS One. 2024 Nov 1;19(11):e0311565. doi: 10.1371/journal.pone.0311565 (PMC11530062; doi:10.1371/journal.pone.0311565)
Supplement: S6 Fig — A) Representative brightfield images of neurons at 20X magnification. Scale bar equals 50 μm. B) qPCR analysis of neuronal genes. C) Immunocytochemistry for neuronal proteins MAP2 and NeuN. Images taken at 20X and 63X. Scale bars equal 50 μm and 25 μm, respectively. D-E) qPCR analysis of D) imprinted genes and E) bi-allelically expressed genes in the 15q locus. For all qPCR data presented, n = 3 biological replicates. RNA expression is presented relative to the parental H9 line. Error bars represent relative min/max calculated with error propagation. Statistical analysis was performed using a t-Test for two samples assuming equal variances. Significance is reported as the results of the two-tailed test. ** = p<0.01, * = p < 0.05. (PDF) [file pone.0311565.s006.pdf]

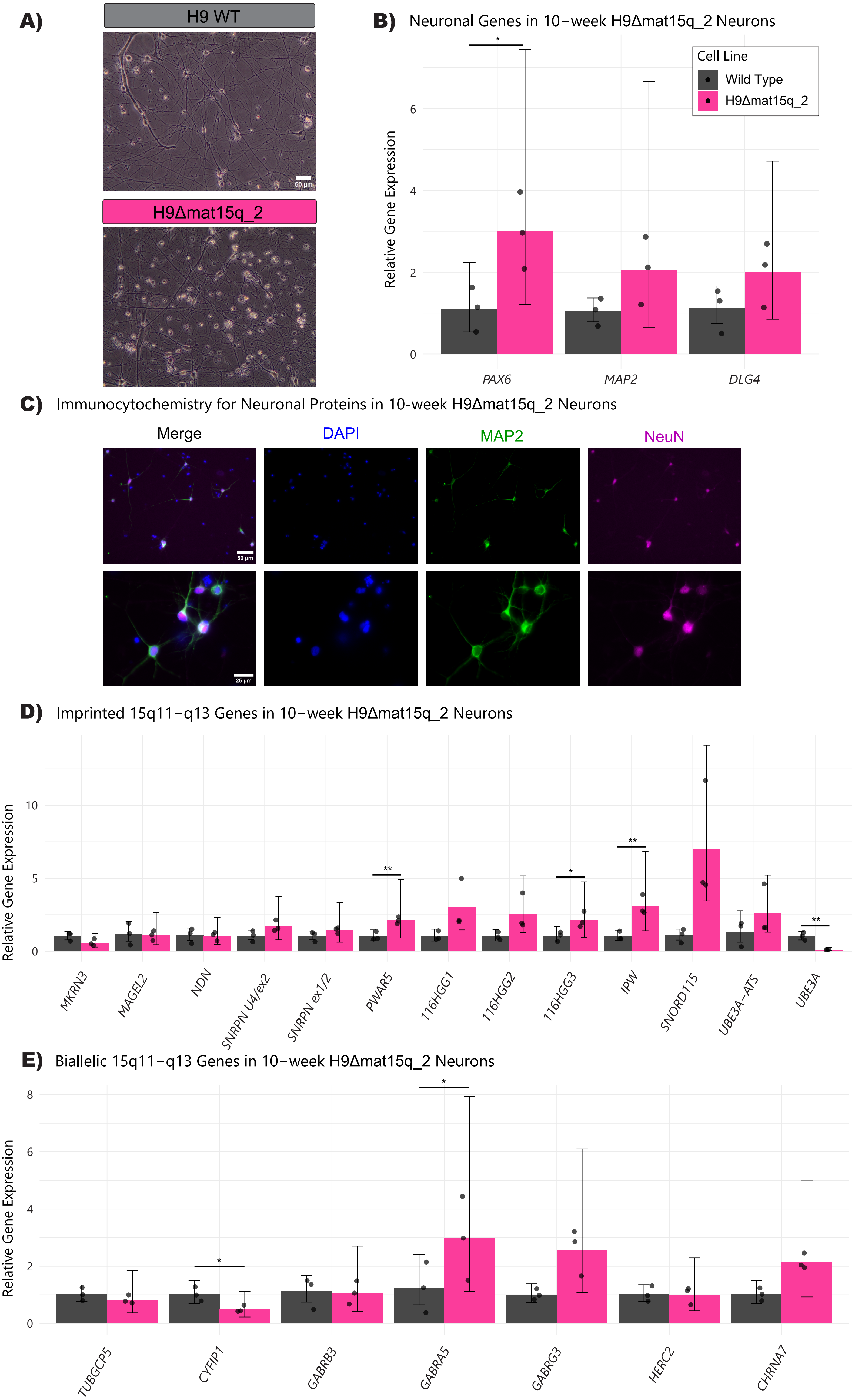

Supplemental Figure 6. Characterization of H9Δmat15q_2 neurons. A) Representative brightfield images of neurons at 20X magnification. Scale bar equals 50 µm. B) qPCR analysis of neuronal genes. C) Immunocytochemistry for neuronal proteins MAP2 and NeuN. Images taken at 20X and 63X. Scale bars equal 50 µm and 25 µm, respectively. D-E) qPCR analysis of D) imprinted genes and E) bi-allelically expressed genes in the 15q locus. For all qPCR data presented, n = 3 biological replicates. RNA expression is presented relative to the parental H9 line. Error bars represent relative min/max calculated with error propagation. Statistical analysis was performed using a t-Test for two samples assuming equal variances. Significance is reported as the results of the two-tailed test. ** = $p<0.01$, * = $p < 0.05$.
