# Supplementary material for: Generation of isogenic models of Angelman syndrome and Prader-Willi syndrome in CRISPR/Cas9-engineered human embryonic stem cells
Source: PLoS One. 2024 Nov 1;19(11):e0311565. doi: 10.1371/journal.pone.0311565 (PMC11530062; doi:10.1371/journal.pone.0311565)
Supplement: S7 Fig — qPCR analysis of A) pluripotency genes and B) neuronal genes in mature 10-week neurons (n = 3 biological replicates). RNA expression is presented relative to the parental H9 line as ESCs. Error bars represent relative min/max calculated with error propagation. Statistical analysis was performed using a one-way ANOVA followed by Dunnett’s test. Significance is reported as the results of Dunnett’s test. **** p<0.0001, *** p<0.001, ** p<0.01, * p<0.05. (PDF) [file pone.0311565.s007.pdf]

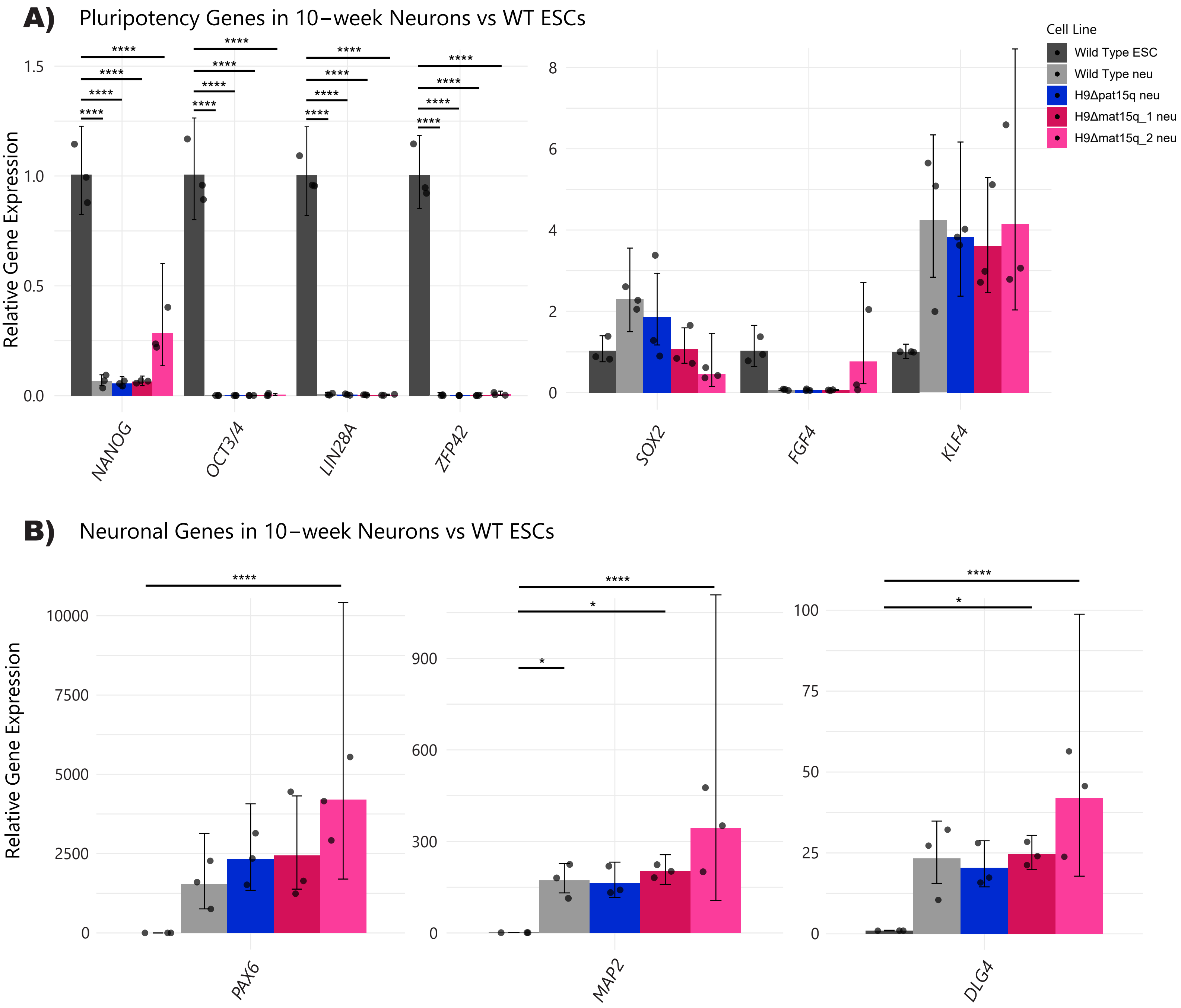

Supplemental Figure 7. Comparative analysis of neurons and ESCs. qPCR analysis of A) pluripotency genes and B) neuronal genes in mature 10-week neurons (n = 3 biological replicates). RNA expression is presented relative to the parental H9 line as ESCs. Error bars represent relative min/max calculated with error propagation. Statistical analysis was performed using a one-way ANOVA followed by Dunnett's test. Significance is reported as the results of Dunnett's test. **** = $p<0.0001$, *** = $p<0.001$, ** = $p<0.01$, * = $p < 0.05$.
