# Supplementary material for: Generation of isogenic models of Angelman syndrome and Prader-Willi syndrome in CRISPR/Cas9-engineered human embryonic stem cells
Source: PLoS One. 2024 Nov 1;19(11):e0311565. doi: 10.1371/journal.pone.0311565 (PMC11530062; doi:10.1371/journal.pone.0311565)
Supplement: S4 Fig — First track displays GOLGA8 CRISPR gRNA binding sites. Second, third, and fourth tracks show deletion contained within each cell line, as determined by CytoSNP. Fifth track displays GENCODEv19 gene annotations; genes in dark blue are protein coding, genes in green are non-coding, arrows indicate direction of gene transcription. Sixth track displays short (<50 bp) variants found in the ClinVar database as a density plot. Seventh track displays ClinVar copy number variants (CNVs); for clarity, only deletions with pathogenic or likely pathogenic annotations are shown. Eighth track shows DECIPHER CNVs, only deletions with pathogenic or likely pathogenic annotations are shown. Ninth track shows conservation scores. Tenth track shows alignment of sequences to other vertebrates. (PDF) [file pone.0311565.s004.pdf]

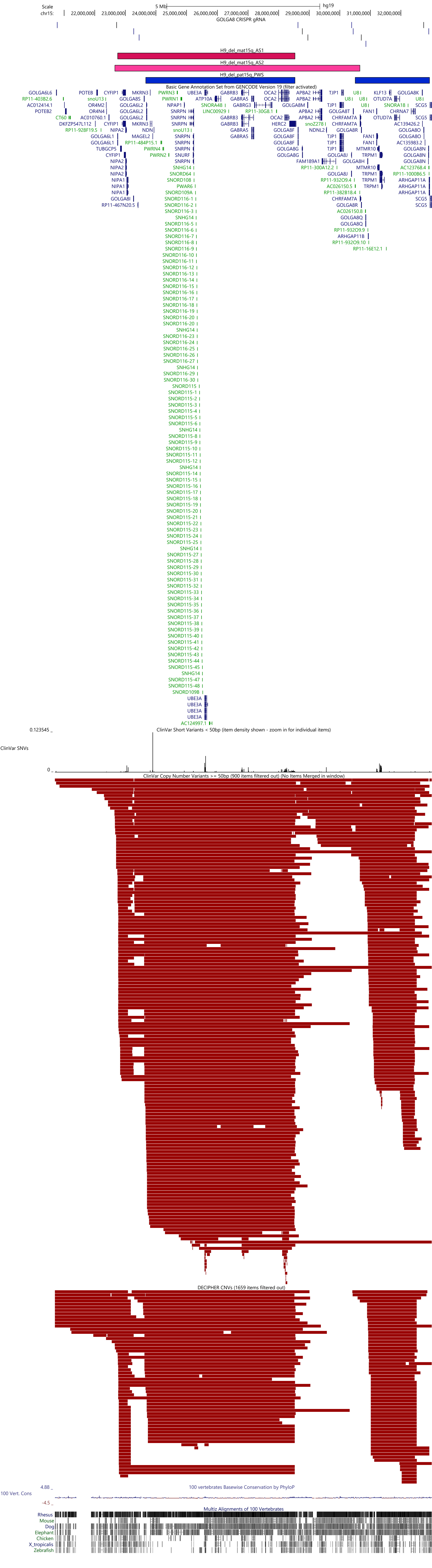

Supplemental Figure 4. UCSC Genome Browser shot of the chromosome 15q locus. First track displays GOLGA8 CRISPR gRNA binding sites. Second, third, and fourth tracks show deletion contained within each cell line, as determined by CytoSNP. Fifth track displays GENCODEv19 gene annotations; genes in dark blue are protein coding, genes in green are non-coding, arrows indicate direction of gene transcription. Sixth track displays short (<50 bp) variants found in the ClinVar database as a density plot. Seventh track displays ClinVar copy number variants (CNVs); for clarity, only deletions with pathogenic or likely pathogenic annotations are shown. Eighth track shows DECIPHER CNVs, only deletions with pathogenic or likely pathogenic annotations are shown. Ninth track shows conservation scores. Tenth track shows alignment of sequences to other vertebrates.
